# Glycolic acid production in the engineered yeasts *Saccharomyces cerevisiae* and *Kluyveromyces lactis*

**DOI:** 10.1186/1475-2859-12-82

**Published:** 2013-09-23

**Authors:** Outi M Koivistoinen, Joosu Kuivanen, Dorothee Barth, Heidi Turkia, Juha-Pekka Pitkänen, Merja Penttilä, Peter Richard

**Affiliations:** 1 VTT Technical Research Centre of Finland, Tietotie 2, Espoo FI-02044, P.O. Box 1000, VTT, Finland

**Keywords:** Glycolic acid, Glyoxylic acid, Metabolic engineering, Glyoxylate cycle, Glyoxylate reductase, *Saccharomyces cerevisiae*, *Kluyveromyces lactis*

## Abstract

**Background:**

Glycolic acid is a C2 hydroxy acid that is a widely used chemical compound. It can be polymerised to produce biodegradable polymers with excellent gas barrier properties. Currently, glycolic acid is produced in a chemical process using fossil resources and toxic chemicals. Biotechnological production of glycolic acid using renewable resources is a desirable alternative.

**Results:**

The yeasts *Saccharomyces cerevisiae* and *Kluyveromyces lactis* are suitable organisms for glycolic acid production since they are acid tolerant and can grow in the presence of up to 50 g l^-1^ glycolic acid. We engineered *S. cerevisiae* and *K. lactis* for glycolic acid production using the reactions of the glyoxylate cycle to produce glyoxylic acid and then reducing it to glycolic acid. The expression of a high affinity glyoxylate reductase alone already led to glycolic acid production. The production was further improved by deleting genes encoding malate synthase and the cytosolic form of isocitrate dehydrogenase*.* The engineered *S. cerevisiae* strain produced up to about 1 g l^-1^ of glycolic acid in a medium containing d-xylose and ethanol. Similar modifications in *K. lactis* resulted in a much higher glycolic acid titer. In a bioreactor cultivation with d-xylose and ethanol up to 15 g l^-1^ of glycolic acid was obtained.

**Conclusions:**

This is the first demonstration of engineering yeast to produce glycolic acid. Prior to this work glycolic acid production through the glyoxylate cycle has only been reported in bacteria. The benefit of a yeast host is the possibility for glycolic acid production also at low pH, which was demonstrated in flask cultivations. Production of glycolic acid was first shown in *S. cerevisiae*. To test whether a Crabtree negative yeast would be better suited for glycolic acid production we engineered *K. lactis* in the same way and demonstrated it to be a better host for glycolic acid production.

## Background

Glycolic acid is the smallest alpha-hydroxy acid containing both alcohol and carboxyl groups. It is used in a wide range of chemical processes, in cosmetic industry and as a precursor for biopolymers. Glycolic acid can be polymerized to polyglycolic acid (PGA), which has excellent gas-barrier properties and mechanical strength, making PGA an ideal packaging material [1]. Glycolic acid is also used together with lactic acid to produce a co-polymer (PLGA) for medical applications, e.g. in drug delivery [2]. Glycolic acid market in 2011 was USD 93.3 million (40 million kg) and expected to reach USD 203 million in 2018 [3].

Currently, glycolic acid is mainly produced chemically from petrochemical resources in a process where toxic formaldehyde is needed. However, from a sustainable point of view a biotechnological production route would be more desirable. Glycolic acid production from lignocellulosic biomass feedstocks would be more sustainable and possibly also economically feasible.

Glycolic acid is naturally produced by a variety of microorganisms from ethylene glycol by oxidation [4] and from glycolonitrile by hydrolyzation [5]. Chemolithotrophic iron- and sulphur oxidizing bacteria are also known to produce glycolic acid by partially unknown metabolic routes in acidic biomining [6]. Since these pathways are either dependent on the availability of ethylene glycol or glycolonitrile or specific environmental conditions, a pathway allowing flexible use of different or more abundant carbon sources under normal bioprocessing conditions would be desirable.

A less explored route is the glyoxylate cycle where C2 compounds such as ethanol are naturally converted into glyoxylate, which can further be converted into glycolic acid by metabolic engineering. Although the main function of the glyoxylate cycle is to ensure the availability of four carbon compounds by utilising two carbon substrates, the naturally existing metabolic routes also allow sugars to be directed to the glyoxylate cycle if the effect of glucose repression of the pathway can be eliminated. The glyoxylate cycle is known to exist in several species within bacteria, fungi and plants. The reactions mostly take place in the cytosol but other cell compartments are also involved [7].

There are a few approaches where the glyoxylate cycle of *Escherichia coli* has been engineered for glycolic acid production [8-12]. However, no eukaryotic microbe has been used for glycolic acid production to the best of our knowledge. With *E. coli*, the fermentation conditions are limited due to their need to be maintained close to a neutral pH. The benefit with many fungal species is their tolerance towards low pH conditions, which is advantageous as the decreased need for neutralisation reduces production costs and the production of waste products. Using eukaryotic microorganisms also has other advantages, such as better inhibitor tolerance for biomass hydrolysates.

In the present study genetically engineered yeast was used to develop a fermentation-based method for glycolic acid production. The work demonstrates that glycolic acid can be produced from the glyoxylate cycle through the reduction of glyoxylate to glycolic acid using a glyoxylate reductase enzyme. *S. cerevisiae* has a gene coding for a NADPH specific glyoxylate reductase, *GOR1*, which is not expressed under normal growth conditions and has a low affinity for glyoxylate [13]. A higher affinity for glyoxylate has been reported for the glyoxylate reductase, GLYR1, from *Arabidopsis thaliana*[14] that is also NADPH requiring. This enzyme, GLYR1, is not naturally part of the glyoxylate cycle in plants, but is involved in the GABA (γ-aminobutyrate) pathway, which catalyses the detoxification of photorespiratory glyoxylate and succinic semialdehyde [15].

**Figure 1:**
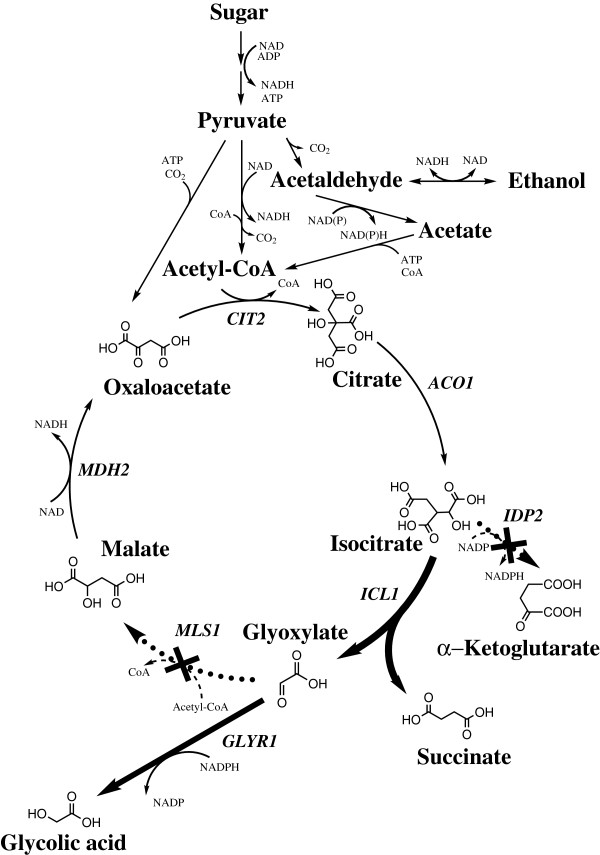
**Engineered glyoxylate cycle.** Engineering of the glyoxylate cycle for glycolic acid production in *Saccharomyces cerevisiae*. The genetic modifications done in this work are indicated by bold and dashed arrows. Production and consumption of redox co-factors on the steps from sugar to pyruvate are dependent on the substrate used as a starting material.

In this communication we describe for the first time yeast strains engineered to produce glycolic acid. Production was demonstrated in *S. cerevisiae* by the expression of a high affinity glyoxylate reductase in combination with a deletion of the two malate synthase encoding genes to prevent glyoxylate conversion to malate. Production of glycolic acid was further increased by the deletion of the gene encoding the cytosolic form of the isocitrate dehydrogenase and overexpression of the isocitrate lyase encoding gene (Figure 1). The deletion of malate synthase gene and the cytosolic form of isocitrate dehydrogenase gene were also carried out in *K. lactis*. The modified *K. lactis* strain produced up to about 15 g l^-1^ of glycolic acid from ethanol and d-xylose in a bioreactor cultivation.

## Results

### Toxicity of glycolic acid

**Figure 2:**
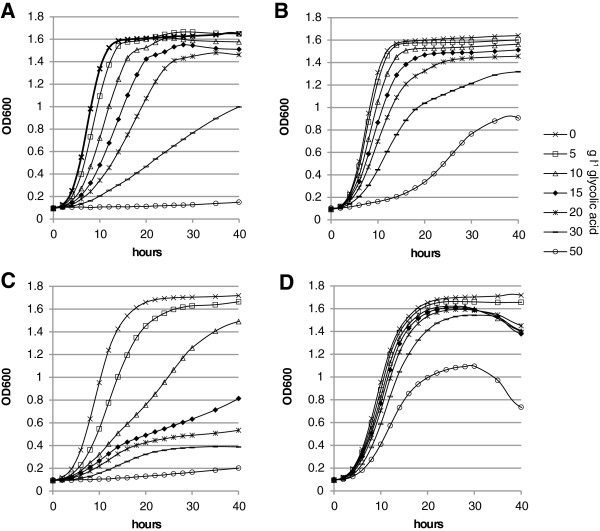
**Toxicity of glycolic acid.** The Bioscreen toxicity assay growth curves of the *Saccharomyces cerevisiae* CEN.PK2-1D strain **A)** at pH 3, and **B)** at pH 5 and *Kluyveromyces lactis* KHO69-8C strain **C)** at pH 3, and **D)** at pH 5. Yeast was grown on SCD medium with different glycolic acid concentrations.

The toxicity of glycolic acid was tested using Bioscreen cultivations where different concentrations of glycolic acid/glycolate were used. In the first experiment with *S. cerevisiae*, the pH was not adjusted and growth stopped at about 10 g l^-1^ glycolic acid (data not shown). We then adjusted the pH to pH 3 or pH 5 at different glycolic acid concentrations (Figure 2). The pKa of glycolic acid is 3.83 meaning that at pH 3 glycolic acid is mainly present in its undissociated form. At pH 3 the growth rate of *S. cerevisiae* is considerably reduced at glycolic acid concentration of 30 g l^-1^ and no growth is observed at 50 g l^-1^. With *K. lactis* the growth is reduced already at glycolic acid concentration of 15 g l^-1^. At pH 5, where glycolic acid is mainly present in its dissociated form, growth is still observed at a concentration of 50 g l^-1^, however at a reduced rate.

### Glyoxylate reductase activity

The activity of the endogenous *S. cerevisiae* glyoxylate reductase *GOR1*[13] and the *A. thaliana GLYR1*[14] he *GLYR1* gene was codon optimised for expression in yeast. Both genes were overexpressed in *S. cerevisiae* from the same multicopy plasmid and the activity with glyoxylic acid and NADPH was measured from crude extracts. For glyoxylic acid, the K_m_ was 1.9 mM and the Vmax 1.8 U (mg total protein)^-1^ for the endogenous Gor1p, and for the *A. thaliana* GLYR1 the K_m_ was 38.8 μM and the Vmax 6.5 U (mg total protein)^-1^.

### Glycolic acid production in *S. cerevisiae*

Glycolic acid production was tested in *S. cerevisiae* strains where either the *GOR1* or *GLYR1* was overexpressed. No glycolic acid production was detected in a strain where the endogenous glyoxylate reductase, *GOR1*, was expressed. Since GLYR1 was found to have a much higher reaction rate and affinity than that of Gor1p and based on the fact that no glycolic acid was produced by overexpressing *GOR1*, we decided to exclusively use *GLYR1* in further strain constructions as illustrated in Figure 1. As a parent strain we used H3675, a modified CEN.PK113-1A strain, which contains a eukaryotic d-xylose utilisation pathway including overexpressed endogenous *S. cerevisiae* xylulokinase encoding gene and genes for d-xylose reductase and xylitol dehydrogenase from *Scheffersomyces* (*Pichia*) *stipitis*[16,17].

To prevent glyoxylic acid from reacting with acetyl-CoA to form malate, the malate synthase gene *MLS1* was deleted. *DAL7*, a homologue of *MLS1*, was also deleted to ensure that there was no malate synthase activity. To confirm this, malate synthase activity was measured from crude cell extracts from cells grown on glycerol-ethanol mixture. In the absence of malate synthase activity, growth on ethanol is not possible and because of this the growth media contained also glycerol. In the parent strain, malate synthase activity was 0.19 U mg^-1^ and in the strains with one (H3675 *mls1-Δ1*) or two (H3675 *mls1-Δ1*, *dal7-Δ1*) malate synthase genes deleted the malate synthase activity was not detectable.

**Figure 3:**
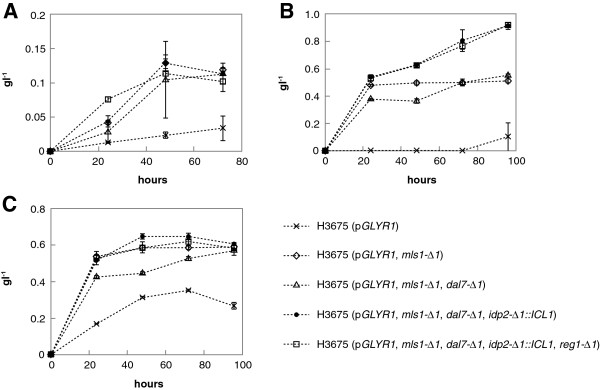
**Glycolic acid production in flask cultivations.** Engineered H3675 based *Saccharomyces cerevisiae* strains were tested for glycolic acid production on different carbon sources in shake flask cultivations on SC media: **A)**d-glucose (20 g l^-1^), **B)**d-xylose (20 g l^-1^) + EtOH (20 g l^-1^), **C)** EtOH. There was no glycolic acid production detected in the parent strain H3675. During the cultivation the pH dropped from pH 5.5 to pH 2.9-3.2. Error bars represent ± the standard error of the mean (SEM; n = 3) and when not visible are smaller than the symbol.

To increase glycolic acid production, the isocitrate lyase encoding gene *ICL1* was overexpressed and the cytosolic form of the isocitrate dehydrogenase encoding gene *IDP2* was deleted. Since the glyoxylate cycle is glucose repressed in *S. cerevisiae*[18], we also deleted the gene for the regulatory factor mediating glucose repression, *REG1*. The resulting strains were cultivated in shake flasks in non-buffered synthetic complete media containing either d-glucose (20 g l^-1^), d-xylose (20 g l^-1^) and ethanol (20 g l^-1^), or only ethanol (20 g l^-1^) as a carbon source. Samples from the cultures were analysed for glycolic acid production using HPLC (Figure 3). Final titers of glycolic acid and biomasses are presented in Table 1.

**Table 1:** **Final titers of glycolic acid and biomasses in *Saccharomyces cerevisiae *flask cultivations**

	**Final titers of glycolic acid (g l**^**-1**^**)**			**Final biomass (g **_**CDW **_**l**^**-1**^**)**		
**Strain**	**d-glucose**	**d-xylose + EtOH**	**EtOH**	**d-glucose**	**d-xylose + EtOH**	**EtOH**
**H3744**	0.03 ± 0.02	0.1 ± 0.10	0.27 ± 0.02	5.62 ± 0.04	8.08 ± 0.06	5.27 ± 0.23
**H3788**	0.12 ± 0.00	0.51 ± 0.02	0.59 ± 0.00	3.33 ± 0.06	4.65 ± 0.15	1.10 ± 0.03
**H3790**	0.11 ± 0.01	0.55 ± 0.01	0.57 ± 0.02	3.65 ± 0.06	4.43 ± 0.12	0.91 ± 0.06
**H3846**	0.11 ± 0.02	0.90 ± 0.01	0.60 ± 0.01	3.70 ± 0.15	4.98 ± 0.04	1.48 ± 0.07
**H3994**	0.10 ± 0.01	0.91 ± 0.02	0.58 ± 0.02	3.78 ± 0.06	5.35 ± 0.23	1.07 ± 0.09

Final titers and final biomasses of different glycolic acid producing *S. cerevisiae* strains grown in SC media containing either d-glucose (20 g l^-1^), d-xylose (20 g l^-1^) and ethanol (20 g l^-1^), or only ethanol (20 g l^-1^) as carbon sources. The d-xylose utilising *Saccharomyces cerevisiae* strain H3675 (*MATα his3 leu2 trp1 ura3::XYL1-XYL2 xks1::XKS1 MAL2-8c SUC2*), a modified [CEN.PK113-1A [50]], was used as a parent strain for all *S. cerevisiae* glycolic acid production strains. ± presents the standard error of the mean (SEM; n = 3).

The expression of the high affinity glyoxylate reductase GLYR1 already resulted in glyoxylate production (Figure 3). Since the glyoxylate cycle is repressed by glucose, we assume that the glucose is first fermented to ethanol, which is subsequently converted to glycolic acid (Figure 3A). To avoid glucose repression, we cultivated the cells in a medium with d-xylose and ethanol (Figure 3B) or only ethanol as carbon sources (Figure 3C). In the medium with only ethanol, cells lacking malate synthase activity were not able to grow. For this reason, cells for all experiments were first pre-grown on d-glucose containing medium, then washed and inoculated at an optical density (OD_600_) of 2 for subsequent flask cultivations for assessment of glycolic acid production.

**Figure 4:**
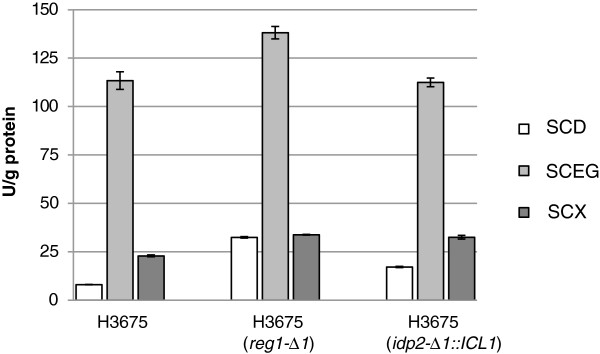
**Effect of *REG1 *deletion on isocitrate lyase activity in presence of different carbon sources.** Isocitrate lyase enzyme activities in the *Saccharomyces cerevisiae* strain when *REG1* is deleted compared to the CEN.PK113-1A based H3675 strain without deletion and to a strain where isocitrate dehydrogenase is deleted and replaced by isocitrate lyase overexpression. SCD = synthetic complete medium with 20 g l^-1^d-glucose, SCEG = synthetic complete medium with 20 g l^-1^ ethanol and 20 g l^-1^ glycerol, SCX = synthetic complete medium with 20 g l^-1^d-xylose. Error bars represent ± the standard error of the mean (SEM; n = 3) and where not visible are smaller than the symbol.

We also tested if more glycolic acid production could be attained by limiting glucose repression by deleting *REG1*. As seen from Figure 3, the *REG1* deletion did not result in increased glycolic acid production in a *S. cerevisiae* glycolic acid production strain H3994 (p*GLYR1*, *mls1-Δ1*, *dal7-Δ1*, *idp2-Δ1*::*ICL1*, *reg1-Δ1*) compared to the similar strain H3846 without *REG1* deletion. We then tested the isocitrate lyase activity in crude cell extracts since the *ICL1* gene is controlled through glucose repression. The parent strain H3675 showed very low isocitrate lyase activity when grown on d-glucose. The *reg1* null mutant showed increased activity on d-glucose (Figure 4) with activity levels being even higher in the *reg1* null mutant strain than when the isocitrate lyase encoding gene was overexpressed. However, independent of the genetic background the highest isocitrate lyase activity was observed when the cells were grown on a mixture of ethanol and glycerol (Figure 4).

### Glycolic acid production in *K. lactis* flask cultivations

**Figure 5:**
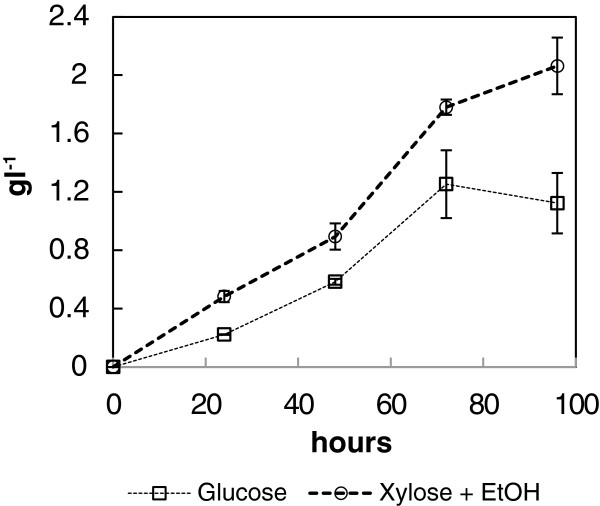
***Kluyveromyces lactis *glycolic acid production in flask cultivations.** Glycolic acid production of *K. lactis* strain H3986 grown in SC media with 20 g l^-1^d-glucose or 20 g l^-1^d-xylose + 20 g l^-1^ EtOH. Error bars represent ± the standard error of the mean (SEM; n = 3) and when not visible are smaller than the symbol.

The high affinity glyoxylate reductase gene *GLYR1* was expressed in *K. lactis* strain containing malate synthase and isocitrate dehydrogenase deletions. These modifications had been analysed to be beneficial for glycolic acid production in *S. cerevisiae* so we assumed that they would increase glycolic acid production also in *K. lactis*. The unmodified *K. lactis* parent strain KHO69-8C did not produce detectable amounts of glycolic acid on d-glucose, d-xylose and ethanol, or only ethanol as a carbon source (data not shown). Similarly to the engineered *S. cerevisiae* strains the glycolic acid production was found out to be the highest when the cells were grown on a mixture of d-xylose and ethanol. The results of the engineered *K. lactis* glycolic acid production strain grown on a mixture of 20 g l^-1^d-xylose and 20 g l^-1^ ethanol (Figure 5) showed that *K. lactis* is a better glycolic acid producer than *S. cerevisiae*.

### Glycolic acid production in *K. lactis* in bioreactor cultivation

**Figure 6:**
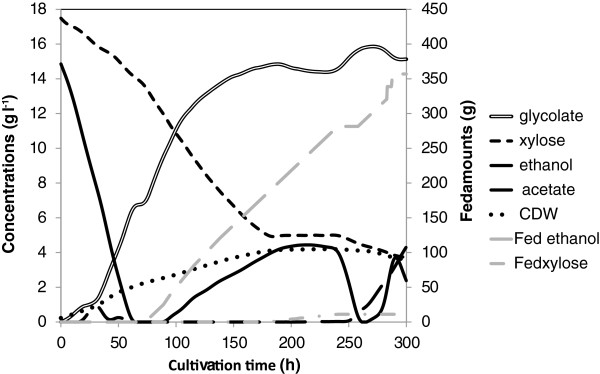
***Kluyveromyces lactis *glycolic acid production in fed-batch bioreactor.** Concentrations (g l^-1^) of glycolate, d-xylose, ethanol, acetate, and cell dry weight (CDW) in fed-batch bioreactor cultivation of *K. lactis* glycolate strain on mixture of d-xylose and ethanol under aerobic conditions at pH 5. Fed amounts (g) of ethanol and d-xylose are also presented. Measurements were performed with on-line HPLC at 0.5-hour intervals and data was smoothed as described in materials and methods.

**Figure 7:**
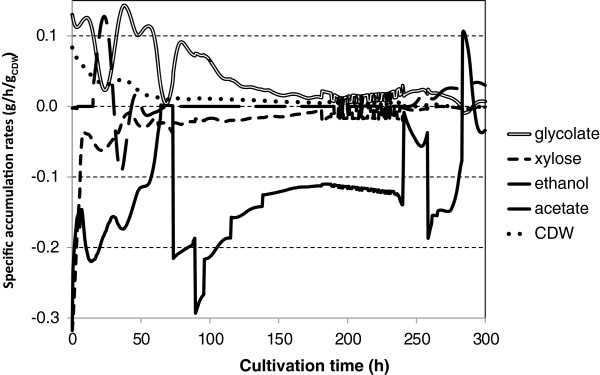
**Specific accumulation rates in fed-batch bioreactor cultivation of *Kluyveromyces lactis.*** Specific accumulation rates (g h^-1^ g^-1^_CDW_) of glycolate, d-xylose, ethanol, acetate and cell dry weight (CDW) in fed-batch bioreactor cultivation of *K. lactis* glycolate strain on mixture of d-xylose and ethanol under aerobic conditions at pH 5. Measurements were performed with on-line HPLC at 0.5-hour intervals and data was smoothed as described in materials and methods.

In order to obtain higher glycolic acid concentrations we cultivated the *K. lactis* strain H3986 also in an aerobic fed-batch bioreactor cultivation at pH 5. We monitored the concentrations of ethanol and d-xylose in the cultivation with on-line HPLC and adjusted their feed automatically after the batch phase in order to try to maintain their concentrations at reasonable levels; with setpoint value for d-xylose above 5 g l^-1^ and ethanol between 1 and 5 g l^-1^. Figure 6 shows the concentrations and Figure 7 shows the specific accumulation rates of the main substrates and products during the cultivation. During the batch phase 6.7 g l^-1^ glycolate was produced in 75 hours. After additional 115 hours with ethanol feed the glycolate concentration increased to 14.8 g l^-1^. The specific accumulation rate of glycolate was the highest in the batch phase between 35 and 60 hours when it stayed above 0.1 g h^-1^ g^-1^ CDW (cell dry weight). During this time the ethanol concentration decreased from 6.7 to 0.4 g l^-1^ and d-xylose concentration decreased from 15.7 to 14.3 g l^-1^. The specific accumulation rate of glycolate was above 0.07 g h^-1^ g^-1^ CDW between 76 and 96 hours while ethanol concentration was below 0.4 g l^-1^ and d-xylose concentration above 11 g l^-1^ and ethanol was fed. Table 2 presents the yields of the *K. lactis* bioreactor cultivation from the beginning of the cultivation to two time points; 95.5 and 167.5 hours. Yields for glycolate and the main side product glyoxylate are calculated based on ethanol consumption and based on a sum of consumption of d-xylose and ethanol. Yields of malate, acetate, cell dry weight and CO_2_ are calculated only based on a sum of ethanol and d-xylose consumption. In all cases the yields were lower at the later time point, which is most likely due to the ethanol evaporation, which has not been taken into account in the calculations. At 95.5 hours the glycolate yield (g g^-1^) from ethanol was above 50%, but also glyoxylate yield was close to 30%. At 167.5 hours the ratio of glycolate vs. glyoxylate improved as 4 times more glyoxylate was produced than glycolate. Minor amounts of malate and acetate were also formed. The volumetric productivity of glycolate at the time points 95.5 and 167.5 hours was 0.11 and 0.09 g l^-1^ h^-1^, respectively. The theoretical maximum yields for glycolic acid production were calculated to be from ethanol 1.00 C-mol / C-mol (1.65 g g^-1^), from d-glucose 0.67 C-mol / C-mol (0.85 g g^-1^), and from d-xylose 0.67 C-mol / C-mol (0.85 g g^-1^). The glycolate yield achieved from ethanol at 95.5 hours, 0.52 g g^-1^, is about 32% of the theoretical maximum yield.

**Table 2:** **Glycolic acid yields in *Kluyveromyces lactis *fed-batch cultivation**

	**Yield (g g**^**-1**^**)**		**Yield (C-mol C-mol**^**-1**^**)**	
	**95.5 h**	**167.5 h**	**95.5 h**	**167.5 h**
Glycolate from ethanol	0.52	0.32	0.31	0.19
Glyoxylate from ethanol	0.28	0.08	0.17	0.05
Glycolate from ethanol and d-xylose	0.44	0.28	0.28	0.18
Glyoxylate from ethanol and d-xylose	0.23	0.07	0.15	0.05
Malate from ethanol and d-xylose	0.005	0.003	0.004	0.002
Acetate from ethanol and d-xylose	0.001	0.000	0.0004	0.0002
CDW from ethanol and d-xylose	0.10	0.06	0.10	0.06
CO_2_ from ethanol and d-xylose	0.32	0.21	0.17	0.11
Sum from ethanol and d-xylose	1.10	0.63	0.70	0.39

Yields of glycolate and the main side products from ethanol and d-xylose from the start until two time-points of *K. lactis* bioreactor fed-batch cultivation.

## Discussion

There are a few patent applications describing glycolic acid production in *E. coli* through the glyoxylate cycle [8-11]. In these approaches glycolic acid production was achieved by overexpressing the endogenous glyoxylate reductases *ycdW* and *yiaE* while attenuating the malate synthase genes *aceB* and *glcB*. Additional modifications were also done such as isocitrate lyase *aceA* overexpression. A very similar approach was recently described by Martin et al. [12] where glycolic acid was used as a precursor for the production of 3,4-dihydroxybutyrate and 3-hydroxy-γ-butyrolactone in *E. coli*, with 1.4 g l^-1^ glycolate production level from 10 g l^-1^d-glucose. In addition, isocitrate lyase and malate synthase encoding genes have been introduced into *Bacillus subtilis*, a species naturally lacking the glyoxylate cycle [19]. This resulted in glycolic acid production although the levels were very low as most of the glyoxylate was presumably converted to malate.

To the best of our knowledge, glycolic acid production has not been attempted in eukaryotic microbes. When using *E. coli* as a production host the fermentation conditions are restricted to neutral pH, while yeast species such as *S. cerevisiae* and *K. lactis* can tolerate acidic pH. This is advantageous since the contamination risk is reduced and the low pH also reduces the need for neutralization. In an industrial process the use of less base would result in less undesired salt formation. Production at acidic pH has been shown to be advantageous for lactic acid [20-22] which is a similar compound to glycolic acid. Lactic acid is a C3 mono-hydroxy acid which has a hydroxyl group adjacent to the carboxyl group like glycolic acid.

Glycolic acid is not very toxic for *S. cerevisiae* or *K. lactis* even at acidic pH. *K. lactis* is more sensitive at pH 3 but less sensitive at pH 5. Both yeast species are able to grow at glycolic acid concentrations up to 30–50 g l^-1^, and thus may be able to produce glycolic acid at high titers.

Essential for the production of glycolic acid from glyoxylic acid is the presence of an active glyoxylate reductase. Glyoxylate reductases can be NADPH dependent (EC 1.1.1.79) or NADH dependent (EC 1.1.1.26). Glyoxylate reductases are found from prokaryotes to mammals but only a few enzymes and the corresponding genes have been characterized. The metabolic role of this enzyme is not unambiguously clear as different functions have been described in different organisms.

In *S. cerevisiae*, two glyoxylate reductase isoforms have been purified [23,24] but only one gene, *GOR1*, encoding glyoxylate reductase activity has been identified [13]. Deletion of the glyoxylate reductase, *GOR1*, led to increased biomass formation after diauxic shift, which was thought to be caused by increased NADPH availability [13]. The biological function of the enzyme is not clear but it may have a role in glyoxylate detoxification as has been described for plants [15].

In plant photorespiration, glyoxylate reductase removes the toxic GABA (γ-aminobutyrate) pathway intermediates glyoxylate and succinic semialdehyde, which can accumulate in plants during stress [15]. In *A. thaliana,* two glyoxylate reductases, GLYR1 [14] and GLYR2 [25] have been characterized. Recent findings have shown that although AtGLYR1 has a peroxisomal targeting signal (PTS1), the protein is exclusively cytosolic suggesting that this particular PTS1 C-terminal tripeptide -SRE is nonfunctonal in plants [26]. The other plant glyoxylate reductase, GLYR2, is found in plastids [14,25]. K_m_ of purified GLYR1 was 4.5 μM for glyoxylate and k_cat_ 13.02 s^-1^[14]. In the present study *GLYR1* was expressed in *S. cerevisiae* the K_m_ of the cell extract being slightly higher, 38.8 μM. The difference in the K_m_ is probably caused by different assay conditions as the buffer and the pH of the reaction mixture were different in these two studies.

In *E. coli*, two genes, *ycdW* and *yiaE*, code for glyoxylate reductase enzymes with the K_m_ being 0.6 mM and 6.6 mM and the k_cat_ being 70.6 s^-1^ and 203 s^-1^ respectively, for the purified proteins [27]. The proteins complement each other but YcdW displays higher affinity for the reduction of glyoxylate whereas YiaE shows higher affinity for the reduction of hydroxypyruvate [27]. These two enzymes have successfully been used for glycolic acid production in *E. coli*[8-12]. We compared the affinities of them and the other glyoxylate reductases, where the encoding gene is known and which are described in the literature [23-25,27-32]. We chose the *A. thaliana* GLYR1 because it had the highest affinity for glyoxylate.

In this work, we showed that overexpression of a glyoxylate reductase gene with low affinity for glyoxylic acid, such as the *S. cerevisiae* Gor1p, did not result in glycolic acid production. However, when expressing a high affinity enzyme such as the GLYR1 from *A. thaliana,* glycolic acid production was observed. The higher affinity of the glyoxylate reductase seems to be crucial for glycolic acid production, at least when no other genetic modifications are made to the host.

The main function of the glyoxylate cycle is to ensure the availability of four carbon compounds during utilisation of two carbon substances as a sole carbon source. The glyoxylate cycle has a similar function when fatty acids are used as a carbon source as beta-oxidation yields two-carbon acetyl-CoA. As a net reaction, the acetyls of two acetyl-CoA molecules are converted to one four carbon compound in the glyoxylate cycle. The C4 intermediate molecule can compensate for the carbon loss of the TCA-cycle.

Several of the enzymes involved in the glyoxylate cycle are isozymes of the enzymes in the TCA cycle. Only isocitrate lyase and malate synthase are found solely in the glyoxylate cycle. Malate synthase, Mls1p, is the main enzyme utilising glyoxylic acid as a substrate in yeast, and its deletion probably resulted in glyoxylic acid accumulation and consequently in improved glycolic acid production. When using ethanol or a d-xylose-ethanol mixture as a carbon source, we observed an increased glycolic acid production rate. However, the lack of functional glyoxylate cycle, e.g. deletion of the malate synthase encoding gene, has the consequence that the cells are no longer able to use C2 carbon sources such as ethanol or acetate for growth. Thus, the dry mass of the cells grown only on ethanol remained constant during the fermentation. To prevent starvation of the cells and to reduce glucose repression, we used d-xylose and ethanol as carbon sources. By using a mixture of these two carbon sources we obtained the highest glycolic acid titers.

In a further attempt to increase the glycolic acid production, we deleted the gene encoding the cytosolic form of the isocitrate dehydrogenase, *IDP2,* while simultaneously overexpressing the isocitrate lyase, *ICL1*, gene. These modifications indeed increased the glycolic acid production but as the modifications were combined it is impossible to further analyse how much of the increase was impact of *IDP2* deletion and which part was influence of *ICL1* overexpression. The overexpression of the *ICL1* using a constitutive promoter resulted in increased isocitrate lyase activity on d-glucose and d-xylose media. However, this activity was much lower than the activity achieved during growth on mixture of ethanol and d-glycerol. This observation - in addition to the role of glucose repression [18,33] - is possibly explained by glucose-induced post-translational modifications such as phosphorylation [34,35] or proteolytic inactivation [36], which have been shown to regulate Icl1p activity.

The glyoxylate cycle enzymes are highly glucose repressed in *S. cerevisiae*. The isocitrate lyase (*ICL1*) [33], aconitase (*ACO1*) [37], citrate synthase (*CIT2*) [38], malate synthase (*MLS1*) [39] and malate dehydrogenase (*MDH2*) [40] enzymes are sensitive to carbon catabolite repression. In *K. lactis* the enzymes of the cycle have not been studied to the same extent but at least isocitrate lyase is similarly glucose repressed as its homolog in *S. cerevisiae* and the deletion of the encoding gene causes the strain to be unable to grow on ethanol media [41]. However, unlike *S. cerevisiae* isocitrate lyase *K. lactis* Icl1p is not subject to carbon catabolite inactivation but the enzyme is regulated only at transcriptional level by glucose repression [41]. Thus, the Icl1p activity responds more rapidly to the addition of fermentable sugars in *S. cerevisiae* than in *K. lactis*.

As many of the enzymes have isozymes participating in different metabolic pathways these parallel enzymes can partially compensate for the defect; however the differences in expression and localization result in only partial compensation in *S. cerevisiae*[7]. Excluding the isocitrate lyase, the other glyoxylate cycle enzymes are known to have isozymes in *S. cerevisiae*. An example are the citrate synthases *CIT1* (TCA-cycle) and *CIT2* (glyoxylate cycle); both need to be disrupted in order to produce citrate synthase deficient strains, which do not grow on non-fermentative carbon sources [38]. There are also two different genes coding for malate synthase: *MLS1* that functions in the glyoxylate cycle and *DAL7* that is part of allantoin catabolism [39]. Both of these genes were deleted in our *S. cerevisiae* production strains but no further increase of glycolic acid production was detected after *DAL7* deletion, leading to the conclusion that the isozyme was not competing with the glyoxylate cycle on the use of cytosolic glyoxylate.

At the transcriptional level, one of the most central regulators of the glyoxylate cycle is the serine/threonine kinase encoded by *SNF1*, which is highly conserved among eukaryotes [42]. Both *S. cerevisiae* and *K. lactis* require the Snf1p kinase together with the Cat8p transcriptional activator for the utilisation of nonfermentable carbon sources [43]. In *S. cerevisiae*, Snf1p forms a protein complex with the activator subunit Snf4p and other proteins like Sip1p, Sip2p and Gal83p [44]. The activity of Snf1p is dramatically increased during glucose starvation and it is repressed on glucose medium. However, in *K. lactis* the Cat8p transcription is not similarly carbon source regulated, which indicates that posttranscriptional regulation of Cat8p has a prominent role in *K. lactis*[43]. Furthermore, the active form of Snf1p regulates the expression levels of several genes encoding key enzymes in carbon metabolism. The protein phosphatase complex Reg1Glc7 of *S. cerevisiae* seems to be a negative regulator of Snf1p while Reg1p functions as a targeting subunit of the phosphatase [45]. In fact, glucose repression does not function anymore in the *reg1* null mutant strain [46]. Nonetheless, the exact mechanism behind glucose repression is still unknown [47].

As shown in this article, deletion of the *S. cerevisiae REG1* gene had a beneficial effect on isocitrate lyase activity in d-glucose and d-xylose grown cells. However, the Icl1p activity of cells grown on ethanol-glycerol mixture was markedly higher. This suggests that the activity is not only repressed through the Reg1p but also other regulatory mechanisms are involved. This is also supported by the observation that glycolic acid production was not increased by the *reg1* mutation.

Unlike bacteria, eukaryotic microorganisms are compartmentalized and the reactions of the glyoxylate cycle take place in different cellular compartments, the cytosol, the peroxisomes and the mitochondria. The latter one is involved through the TCA cycle, which is closely associated with the glyoxylate cycle through many shared metabolites. Differences occur between eukaryotic organisms related to the localisation of the enzymes in cytosol and peroxisomes [7]. Current knowledge suggests that the *S. cerevisiae* isocitrate lyase (Icl1p), aconitase (Aco1p) and malate dehydrogenase (Mdh2p) are located in the cytosol and citrate synthase (Cit2p) and malate synthase (Mls1p) are peroxisomal enzymes. However, despite the presence of a peroxisomal targeting signal on Mls1p, it can be localised to the cytosol during growth on ethanol or acetate medium [7]. The reason for these two different localizations is probably due to the availability of acetyl-CoA. During ethanol or acetate catabolism, acetyl-CoA is produced in the cytoplasm while the beta-oxidation is carried out inside the peroxisomes [48].

Like Mls1p, Cit2p also has a peroxisomal targeting signal (SKL) but in contrast to the mixed localization of Mls1p, Cit2p is found only in peroxisomes. This fact suggests that peroxisomal Cit2p is not capable of utilising cytosolic acetyl-CoA in the glyoxylate cycle. In fact, the *cit2* null mutant strain does not display any particular phenotype whereas the *cit1* null mutant strain cannot grow on acetate medium [49]. This observation indicates that when cytosolic acetyl-CoA from ethanol or acetate catabolism is utilised in the glyoxylate cycle, the citrate synthase activity might come from the mitochondrial Cit1p. Of the enzymes directly involved in glycolic acid production, citrate synthase is the only one located outside the cytosol. Thus, transport is required between the different cell compartments making the yeast production route more complicated compared to the bacterial glyoxylate cycle.

## Conclusions

In this communication we describe for the first time a yeast strain that was engineered to produce glycolic acid through the glyoxylate cycle. In the present work, up to 15 g l^-1^ of glycolic acid was produced by the engineered yeast. In flask cultivations the production was shown to be possible also at a pH where the acid is in its undissociated form. For the economical production of glycolic acid, the titer still has to be improved. However, this is a successful first demonstration of glycolic acid production at low pH.

## Methods

### Yeast strains

The *S. cerevisiae* strain H3675 (*MATα his3 leu2 trp1 ura3::XYL1-XYL2 xks1::XKS1 MAL2-8c SUC2*), which is a d-xylose utilising modification of VTT C-10880, [CEN.PK113-1A, kindly provided by Dr. P. Kötter (Institut für Mikrobiologie, J.W. Goethe Universität Frankfurt, Germany) [50], was used as a parent strain for all further *S. cerevisiae* modifications. The strain has been modified to utilise d-xylose by introducing *XYL1* (d-xylose reductase) and *XYL2* (xylitol dehydrogenase) genes and by overexpression of the endogenous *XKS1* (xylulokinase) gene [16,17]. The *K. lactis* strain H3954 [KHO69-8C (*MATα ura3 leu2 his3::loxP ku80::loxP*)] [51] was used as a parent strain for all further *K. lactis* modifications. All strains constructed in this work are listed in Table 3.

**Table 3:** Glycolic acid production strains constructed in this work

**Strain number**	**Modifications**	**Auxotrophies**
H3744	*S. cerevisiae* H3675 (p*GLYR1*)	*his3, leu2, trp1*
H4241	*S. cerevisiae* H3675 (*reg1-Δ1*)	*his3, leu2, trp1, ura3*
H4242	*S. cerevisiae* H3675 (*idp2Δ::ICL1*)	*his3, leu2, trp1, ura3*
H3788	*S. cerevisiae* H3675 (p*GLYR1*, *mls1-Δ1*)	*his3, leu2, trp1*
H3790	*S. cerevisiae* H3675 (p*GLYR1*, *mls1-Δ1*, *dal7-Δ1*)	*his3, leu2, trp1*
H3846	*S. cerevisiae* H3675 (p*GLYR1*, *mls1-Δ1*, *dal7-Δ1, idp2-Δ1*::*ICL1*)	*his3, leu2, trp1*
H3994	*S. cerevisiae* H3675 (p*GLYR1*, *mls1-*Δ1, *dal7-Δ1*, *idp2-Δ1*::*ICL1, reg1-Δ1*)	*his3, leu2, trp1*
H3968	*K. lactis* H3954 (*mls1-Δ1)*	*ura3 leu2 his3*
H3976	*K. lactis* H3954 (*mls1-Δ1, idp2-Δ1)*	*ura3 leu2 his3*
H3986	*K. lactis* H3954 (*pGLYR1*, *mls1-Δ1*, *idp2-Δ1*)	*leu2 his3*

The xylose utilising *Saccharomyces cerevisiae* strain H3675 (*MATα his3 leu2 trp1 ura3::XYL1-XYL2 xks1::XKS1 MAL2-8c SUC2*), a modified VTT C-10880, [CEN.PK113-1A [50]], was used as a parent strain for all *S. cerevisiae* glycolic acid production strains. The *Kluyveromyces lactis* strain H3954 [KHO69-8C (*MATα ura3 leu2 his3::loxP ku80::loxP*)] [51] was used as a parent strain for all *K. lactis* glycolic acid production strains.

### Engineering of the *S. cerevisiae*

The glyoxylate reductase gene of *A. thaliana*, *GLYR1*, was codon optimised for *S. cerevisiae* by GenScript (GenScript, USA). For heterologous expression in *S. cerevisiae,* the plasmid was digested with *Eco*RI and *Bam*HI (both NEB) and the *GLYR1* gene was ligated between the *Eco*RI and *Bam*HI sites of p2159, which was derived from the pYX212 plasmid (R&D Systems) as described in Verho et al. [52]. This plasmid has a constitutive *TPI1* promoter and *URA3* for selection. The yeast strain H3675 was transformed with the plasmid and the transformants were selected for URA prototrophy. All yeast transformations were done using the lithium acetate method [53].

The pUG6 plasmid [54] with a *KanMX* marker gene and *loxP* sites was used as a template for malate synthase, *MLS1*, deletion. The deletion cassette was prepared by PCR using primers O8635 and O8636 (all oligo sequences are listed in Table 4) which both consisted of about 40 bp of flanking region for homologous recombination upstream and downsteam of the *S. cerevisiae MLS1* gene. For *DAL7* deletion, a similar plasmid p1431 with a *BLE* marker gene [55] was used. Primers O3637 and O3638 both consisted of about 40 bp of flanking region for homologous recombination upstream and downsteam of the *S. cerevisiae DAL7* gene. Transformants were placed on YPD plates supplemented with 200 μg ml^-1^ of G418 (Invitrogen, USA) or 100 μg ml^-1^ of Zeocin (Invitrogen, USA) for selection. Deletions were checked by yeast colony-PCR with primers O8639, O8640 (*MLS1*) and O8641, O8642 (*DAL7*). The *REG1* deletion was carried out in a similar manner as the *MLS1* deletion but with the primer pair O8661 and O8662. All deletion cassettes were prepared using the Phusion polymerase (Thermo Scientific).

**Table 4:** Primers used in this work

**Number**	**Sequence 5′ → 3′**
O8635	*TTCTACACTGGCTACCGATTTAACTCATCTTCTTGAAAGT*CTGCAGGTCGACAACCCTTA
O8636	*TCATGATAAGATGATTCATTGCTAACTACGAAACGAAGCA*CACTAGTGGATCTGATATCACCT
O8637	*GAGTTCAATTTGTCATACCTTATCAGGCCTTATCAGCTCA*CTGCAGGTCGACAACCCTTA
O8638	*TGCAGTTGATATCACTTAGAGTATGTGTCATAGGCACGGT*CACTAGTGGATCTGATATCACCT
O8639	TGCAGTGTCAGCCTTACGA
O8640	CGTTGCTATACTTTCTCGAC
O8641	CAAGGACACCGTCATTGAC
O8642	CATAGGTTTACGATTATCGAC
O8649	*CTTTTCTTGCTTAAATCTATAACTACAAAAAACACATACA*ATGCCTATCCCCGTTG
O8650	*CATTGTTCCTTATTCAGTTAGCTAGCTGAGCTCGACTCTAG*CTATTTCTTTACGCCATTTTC
O8651	CTATTTTCCCTTCTTACG
O8652	CGTTCATTGTTCCTTATTC
O8653	*CCAGCTGGCGAAAGGGGGATGTGCTGCAAGGCGATTAAGT*GAATTGGGGATCTACGTATG
O8654	*TACATTATACGAAGTTATATTAAGGGTTGTCGACCTGCAG*CGAATTGGAGCTAGACAAAG
O8655	*ATACATCCCTTTTTTTTTTTGTCTTTGTCTAGCTCCAATTCG*CTGCAGGTCGACAACCCTTA
O8656	*ACCCTCACTAAAGGGAACAAAAGCTGGAGCTCCACCGCG*CACTAGTGGATCTGATATCACCT
O8657	CTAAATCAATCTTTTTCAATTT
O8658	CATACATTATACGAAGTTATATTAAGG
O8659	*CCAGACTAATGATCAAGTAACTGTGGATTCTGCCACCGCGA*GAATTGGGGATCTACGTATG
O8660	*CTTTTTTCAATCTAGATTCCACCGCGTCAATGAACTCCTCGG*CACTAGTGGATCTGATATCACCT
O8661	*ATGTCAACAAATCTAGCAAATTACTTCGCCGGTAAGAAAG*CTGCAGGTCGACAACCCTTA
O8662	*CTAACTGCTGTCATTTCCATTTTCTTGTGGCTTGACGTCA*CACTAGTGGATCTGATATCACCT
O8643	GTCGACGATCTACGTATGGTCATTTCTTC
O8644	GTCGACCGAATTGGAGCTAGACAAAG
O8768	*CAATGAGGCTTTCTTAAGTTATGCAAGCTGTGTGTAGAGTCGTCATCCCT*GCAGGTCGACAACCCTTAAT
O8769	*GGATAAAAGCTCTATACAGACTACTATCAGAAAACTTTATTAAAGATTCA*GGCCACTAGTGGATCTGATA
O8772	*GATACTCAACGCAGTTAAGCATCTTTAGCAATCAAATTTAGCAGCCCATT*GCAGGTCGACAACCCTTAAT
O8773	*GGATATATAATGTAGCAGTAAATCGGATAGGGAAAGGCTAGCTTTCTTCT*GGCCACTAGTGGATCTGATA

Sequences in underlined italics show the flanking sequence needed for homologous recombination in the yeast transformation.

Yeast DNA for yeast colony PCR was extracted by incubating a single colony in 50 μl of 1 mg ml^-1^ Zymolyase 100 T (Seikagaku Biobusiness, Japan) in 1 M sorbitol for 10 minutes at room temperature. After incubation the extract was centrifuged for 15 seconds at 2000 *g*. The supernatant was discarded and the remaining pellet was heated at +95°C for 5 minutes. The dry pellet was resuspended into 30 μl water and 10 μl of it was used as a template in PCR. All colony PCR reactions were carried out with the DyNAzyme II polymerase (Thermo Scientific).

To catalyse the cleavage of the *loxP*-sites, the Cre recombinase was expressed in *S. cerevisiae* by transforming the Cre expression plasmid pSH47 including the *URA3* selection gene [54] into the yeast. The Cre recombinase gene is under control of the galactose inducible promoter, which was activated by growing the transformants for one to two days in liquid media containing 20 g l^-1^ galactose as a carbon source. After the ability to grow on the named antibiotic selection was lost, the pSH47 plasmid and ability to grow on uracil deficient media was lost by growing the strain in rich liquid media (YPD).

The expression of isocitrate lyase, *ICL1*, and deletion of isocitrate dehydrogenase, *IDP2*, were carried out by a single integration cassette. The cassette was prepared by homologous recombination in yeast as described by Colot et al. [56]. The *ICL1* gene was first amplified by PCR using genomic DNA from the H3675 strain as a template. The primers O8649 and O8650 used for the amplification included flanks for homologous recombination with plasmid p2159. The *S. cerevisiae* FY834 [57] strain was transformed with the resulting *ICL1* fragment together with the *Eco*RI and *Bam*HI (both NEB) digested p2159 plasmid for homologous recombination. The transformed yeast cells were cultured on synthetic complete media (SC) (modified from Sherman, 1983) [58] with 20 g l^-1^d-glucose (SCD) plates lacking uracil. Recombined plasmids were extracted from yeast and amplified in *E. coli*. In order to find the correct plasmid constructs, colony PCR was carried out with the primers O8651 and O8652. The *ICL1* overexpression fragment was then amplified from the correct p2159-*ICL1* plasmid by PCR with primers O8653 and O8654. The primer pair included about 40 bp flanking region for homologous recombination with the *loxP*-*KanMX*-*loxP* fragment and pRS426 shuttle vector [59].

The *loxP*-*KanMX*-*loxP* fragment was amplified by PCR with primers O8655 and O8656 using the pUG6 plasmid [54] as a template. The primer pair has flanking regions for homologous recombination with the previously constructed *ICL1* overexpression fragment and with the pRS426 shuttle vector. For constructing the *idp2Δ::ICL1* cassette, the *S. cerevisiae* CEN.PK2-1D strain (*MATα ura3-52 leu2-3*,*112 trp1-289 his3Δ MAL2-8*^*c*^*SUC2*) [60] was transformed with the *Eco*RI and *Xho*I (both NEB) digested pRS426 plasmid*, ICL1* overexpression fragment and *loxP*-*KanMX*-*loxP* fragment. The plasmid that was derived through homologous recombination was rescued from yeast and amplified in *E. coli*. The correct plasmid composition was confirmed by colony PCR and sequenced with primers O8657 and O8658. The *idp2Δ::ICL1* cassette was amplified from the correct plasmid with primers O8659 and O8660 including the flanking regions for homologous recombination into the *IDP2*-locus of *S. cerevisiae*. The fragment was transformed to the H3790 strain where the *MLS1* and *DAL7* genes had been deleted as previously described. G418 antibiotics were used for selection and an enzyme assay was used to check the functionality of isocitrate lyase overexpression. The looping out of the *KanMX* marker was done with Cre recombinase as described above.

### Engineering of the *K. lactis*

The expression vector for *K. lactis*, pJJH958r [51] was used as a backbone for *GLYR1* glyoxylate reductase gene overexpression. The *S. cerevisiae* codon optimised *A. thaliana GLYR1* gene was also used for *K. lactis* constructs. The pJJH958r plasmid was digested with *Kpn*I to remove the *CreR* gene with *GAL1* promoter and *CYC1* terminator and the vector was then religated. The resulted circular vector, p4150, was digested with *Sal*I. The *GLYR1* gene with the *TPI1* promoter and a terminator was amplified by PCR using primers O8643 and O8644 to introduce *Sal*I restriction sites. The PCR fragment was digested with *Sal*I and ligated to the *Sal*I site of the p4150 vector. The resulting multicopy vector expressing *GLYR1* gene, p4185, has the *URA3* gene as a selection marker.

The *K. lactis* gene [Gene ID: 2895299] was identified as the gene with the highest homology to the *S. cerevisiae MLS1* and was deleted following a protocol described previously [51]. The *HIS3* gene of the plasmid pJJH955H [51] was amplified by PCR using primers O8768 and O8769 that had 50 bp flanking regions upstream and downstream the open reading frame of the *MLS1* homologue. The PCR product was then transformed to the *K. lactis* strain H3954 and transformants selected for the ability to grow in the absence of histidine. The deletion cassette was looped out as described previously [51]. The success of the deletion was confirmed by testing the growth on ethanol media.

The *K. lactis* gene with the [Gene ID:2894935] was identified as the closest homologue to the *S. cerevisiae IDP2* gene. It was deleted in the H3968 in a similar way as the *MLS1* homologue described above using the primers O8772 and O8773. Deletions were checked by yeast colony-PCR. The resulting strain H3976 was transformed with the *GLYR1* expression vector, p4185, resulting in the strain H3986. The strain was tested for glycolic acid production.

### Toxicity of glycolic acid

To test the toxicity of glycolic acid to yeast cells, the growth of the *S. cerevisiae* CEN.PK2-1D (*MATα ura3-52 leu2-3*,*112 trp1-289 his3Δ MAL2-8*^*c*^*SUC2*) [60] and *K. lactis* KHO69-8C strains were monitored in a computer-controlled Bioscreen C MBR incubator (Oy Growth Curves Ab Ltd, Finland) in different glycolic acid concentrations varying between 0 – 50 g l^-1^. Cells were precultured overnight on SCD and diluted to the final OD_600_ of 0.5 with water. 30 μl of the cell suspension was added into each well of the Bioscreen plate containing SCD medium and the desired amount of glycolic acid (Sigma-Aldrich, Germany). Total volume of each well was 300 μl. The pH was either not adjusted or it was adjusted with NaOH to pH 3 or to pH 5. Plates were incubated in the Bioscreen at 30°C for 40 hours and the OD_600_ was measured at 30-minute intervals.

### Enzyme assays

For the protein extractions, *S. cerevisiae* strains were cultured overnight on YPD (20 g l^-1^ Bacto-peptone (BD, USA), 10 g l^-1^ Bacto-yeast extract (BD, USA), 20 g l^-1^d-glucose). Washed cells from each culture were resuspended into 5 ml of 10 mM sodium phosphate buffer with EDTA free protease inhibitor (Roche, USA). The cells were disrupted three times for 30 sec in a Fast Prep (Bio 101). The cell extracts were centrifuged (15 min, 25000 *g*, 4°C) and the supernatants were used in the assay.

The following reaction mixture was used for assaying the glyoxylate reductase activity in strains expressing *GLYR1*: 50 mM sodium phosphate buffer, pH 7, 200 μM NADPH and glyoxylic acid (Sigma-Aldrich, Germany) where pH was adjusted to ~7.0 with NaOH in concentrations ranging from 2 μM to 90 mM. The consumption of NADPH in the reactions was monitored by measuring absorbance at 340 nm. The reaction was assayed at 25 seconds intervals for 300 seconds after addition of glyoxylic acid. The enzyme assays were performed at 30°C in an Arena 20XT (Thermo, Vantaa, Finland) automated analyser. A Michaelis-Menten-term was fit to the data and the K_m_ values of GLYR1 and Gor1p for glyoxylate were calculated using the GraphPad Prism 5.03 software. Protein concentrations were determined using the Protein Assay Kit (BioRad Laboratories, Hercules, CA, USA).

For the malate synthase (Mls1p and Dal7p) assay, the *S. cerevisiae* strains were precultured overnight on YPD medium and then shifted into SC medium with d-glucose (D) or ethanol and glycerol (EG) as a carbon source. The concentration of each carbon source in the media was 20 g l^-1^. The initial OD_600_ value of each SCD culture was 0.5 while the corresponding value for the SCEG cultures was 1.0. After 7 hours, the cells were lysed as described above except that the sodium phosphate buffer was now replaced by 50 mM potassium phosphate buffer pH 7.0.

The reaction mixture in the malate synthase assay was: 100 mM Tris pH 8.0, 5 mM MgCl_2_, 100 μM acetyl-CoA, 10 μl cell extract and water to 980 μl. After the stabilization, 20 μl glyoxylate was added into the final concentration of 2 mM and the decreasing absorbance of acetyl-CoA at 232 nm was monitored for 6 minutes. The UltrospecTM 2100 pro spectrophotometer (GE Healthcare Life Sciences, USA) and UV quartz cuvettes were used for the malate synthase assay.

For the isocitrate lyase (Icl1p) assay, *S. cerevisiae* strains were precultured overnight on YPD medium and then shifted to SC medium with d-glucose (D), d-xylose (X) or ethanol and glycerol (EG) as a carbon source as described in the MLS-assay. The OD_600_ values of each culture at the beginning were the following: SCD 0.5, SCX 1.5 and SCEG 1.0. After 7 hours, the cells were lysed as described above except the extraction buffer 25 mM Hepes (Sigma-Aldrich, Germany), 1 mM DTT, 100 mM KCl and 1x EDTA free protease inhibitor (Roche, USA) was used.

Isocitrate lyase activities were measured by following the protocol described by Jean Marie Francois’ lab [61] where the two step reaction is monitored at 324 nm. DL-isocitric acid trisodium salt (Sigma-Aldrich, Germany), phenylhydrazine (Sigma-Aldrich, Germany) and buffer (100 mM imidazole (Sigma-Aldrich, Germany), 200 mM KCl, 2 mM EDTA (Sigma-Aldrich, Germany), 10 mM MgSO_4_) were used in the assay. The changes in absorbance were detected for 10 minutes from the beginning of each reaction by using the Varioskan (Thermo Scientific, USA) spectrophotometer. Three replicates were analysed for each strain-medium combination.

### Flask cultivations for glycolic acid production

Cultivations for testing the glycolic acid production were done using a 50 ml volume in 250 ml flasks. The cultivations were started from overnight cultures grown in liquid SCD-URA. The initial OD_600_ was 2.0 ± 0.3 (corresponding to about 0.7 g l^-1^ of cell dry mass) and flasks were shaken with 250 rpm in 30°C. Synthetic complete (SC) media was used with different carbon sources. 20 g l^-1^d-glucose (SCD), 20 g l^-1^ ethanol (SCE) and a mixture of 20 g l^-1^ ethanol and 20 g l^-1^d-xylose (SCEX) were used and three parallel cultivations of each strain and carbon source were done. 2 ml samples were taken daily and the OD_600_, pH and dry mass were measured.

### Fed-batch bioreactor cultivation for glycolic acid production

*K. lactis* strain H3986 was pregrown in shake flasks in modified synthetic complete (SC) medium lacking uracil [58]. d-Glucose (20 g l^-1^) was provided as carbon source. Pregrown cells were used for yeast cultivation started at OD_600_ = 1 in a Biostat CT-DCU bioreactor (max. working volume 5000 ml, Sartorius, Göttingen, Germany) at pH 5.0, 30°C, 1 volume air [volume culture]^-1^ min^-1^ (vvm) and 500 rpm agitation with rushton turbines. The pH was maintained constant by addition of 2 M NaOH. Silicone antifoaming agent (BDH, 0.2 ml l^-1^) was added to prevent excess foaming. Batch medium was SC medium lacking uracil with 20 g l^-1^d-xylose and 15 g l^-1^ ethanol. d-Xylose and ethanol were fed to the bioreactor as separate feeds; 30% ethanol and 4% d-xylose feed. The d-xylose feed contained additionally SC medium lacking uracil. Bioreactor exhaust gas was measured using Innova 1313 Fermentation monitor and Innova 1309 Multipoint sampler (Innova Air Tech Instruments A/S, Ballerup, Denmark). Cell density was measured using Trucell in situ probe (Finesse, San Jose, CA, USA). d-Xylose, ethanol, acetate, glycolate, xylitol and glycerol were measured automatically every 30 minutes using on-line HPLC (Online HPLC Oy, Helsinki, Finland) [62]. The column used in the on-line HPLC analysis was Transgenomic IC-SEP at 55°C with 5 mM H_2_SO_4_ (Merck KgaA, Germany) as an eluent at flow rate of 0.8 ml min^-1^.

### Off-line analyses of the cultivation samples

Dry cell masses were determined by washing the remaining pellet after centrifugation and drying it at +100°C overnight. Tubes were weighed with a Mettler Toledo analytical balance (Mettler Toledo, USA). The culture supernatant samples were analysed with Waters Alliance 2690 HPLC system (Waters, Milford, USA) where the injection volume was 20 μl. An Aminex HPX-87H Organic Acid Column (300 mm × 7.8 mm) (Bio-Rad, USA) linked to a Fast Acid Analysis Column (100 mm × 7.8 mm) (Bio-Rad, USA) was used as a stationary phase in the HPLC. Columns were maintained at +55°C and 2.5 mM H_2_SO_4_ (Merck KgaA, Germany) was used as an eluent with the flow rate of 0.5 ml min^-1^. Waters 2487 dual wavelength UV (210 nm) detector (Waters, Milford, USA) and Waters 410 differential refractometer (Waters, Milford, USA) were used for the detection of glycolic acid, d-xylose, ethanol, acetate and glycerol. Furthermore, the analysis of glycolic, malic and glyoxylic acids were also performed with P/ACE MDQ capillary electrophoresis system (Beckmann Coulter Inc., Fullerton, CA, USA) using UV detection according to Turkia et. al. [63]. In short, background electrolyte solution was prepared from 20 mM 2,3-pyridinedicarboxylic acid, 0.3 mM myristyltrimethylammonium hydroxide, 30 mg l^-1^ Ca^2+^ and 30 mg l^-1^ Mg^2+^ in methanol:water (10:90, v/v). The pH of the solution was adjusted to 9.0 with ammonia. Untreated fused silica capillaries with inner diameter of 50 μm and total length 80 cm (70 cm to the detector) were used. Samples were injected by using 0.5 psi pressure for 15 s. Separation was carried out at −20 kV (reversed polarity) at constant capillary temperature 25°C. Indirect UV detection was set at 254 nm.

### Calculations and smoothing of the measurement data

All measurement data was smoothed appropriately and interpolated to a common time vector. Smoothing method of choice was a cubic spline smoothing algorithm in Matlab (The MathWorks, version R2010a) with smoothing parameters adapted for the respective measurement series.

In order to calculate the real production and consumption rates r_c(t) cleared of dilution effects from the feed, the equation dc(t)/dt = r _ c(t) + Feed(t)/vol(t)(c _ f–c(t)), describing the apparent concentration changes dc/dt in a fed-batch cultivation, was used.

The parameter c(t) describes the measured concentrations of metabolites and biomass, respectively; dc/dt are the apparent changes in concentrations and are calculated by computational derivation of the smoothed and interpolated measurement concentrations; Feed(t) denotes the feed with the concentration c_f of the fed substrate and vol(t) the momentary volume of the culture.

The equation was rearranged to solve for the rates r _ c(t) = dc(t)/dt ‒ Feed(t)/vol(t)(c _ f–c(t)).

The total feed Feed(t) consists of d-xylose feed, ethanol feed as well as base and acid addition by the pH controller. Therefore, in order to calculate the correct concentration of a certain compound in the feed, the equation $\begin{array}{cc} c\_f=c\_\mathrm{fc} & \mathrm{Feed}\_c\left(t\right)/\mathrm{Feed}\left(t\right) \end{array}$ was used, with c_fc the concentration in the particular feed Feed_c(t) and Feed(t) the momentary sum of all feeds.

Since the online HPLC used a filter to recirculate the biomass from the samples taken, the biomass was both diluted by the feed and concentrated by the filter. This was taken into account by an additional negative feed in the biomass equation, equal to the volume loss by online HPLC sampling, with the feed biomass concentration equal to 0.

Specific rates were then calculated by dividing the rates r_c(t) by the smoothed biomass measurements. Computational integration of the consumption or production rates, respectively, gave the accumulated amounts used or produced by the biomass during the fermentation, normalized to 1 liter. The accumulated amounts at the end of the cultivation were used for yield calculations.

## Competing interests

Authors OMK, JK, PR and MP have a patent application on glycolic acid production in eukaryotic microbes.

## Authors’ contributions

OMK carried out the Bioscreen experiments of the study and analysed the data. JK and OMK carried out the design and construction of *S. cerevisiae* strains. PR and OMK designed and constructed the *K. lactis* strains. OMK carried out the flask cultivations of the *S. cerevisiae* and *K. lactis* strains and analysed the data. JPP participated in designing the bioreactor cultivations. JPP and DB developed the on-line HPLC based measurement and control system of the feeds in bioreactor cultivations. DB analysed the data of the online-HPLC samples and did the rate calculations and HT preformed the CE studies and analysed the data. OMK, PR and JPP drafted the manuscript. PR and MP participated in the design and coordination of the study. All authors read and approved the final manuscript.

## References

[B1] Robertson GL Food Packaging 2012 Principles and Practice: Third ed. CRC Press

[B2] Fredenberg S Wahlgren M Reslow M Axelsson A The mechanisms of drug release in poly(lactic-co-glycolic acid)-based drug delivery systems—a review Int J Pharm 2011 415 1–2 34 52 21640806 10.1016/j.ijpharm.2011.05.049

[B3] Glycolic Acid Market - Global Industry Analysis, Size, Share, Growth, Trends and Forecast, 2012–2018 http://www.academia.edu/4251027/Glycolic_Acid_Market_-_Global_Industry_Analysis_Size_Share_Growth_Trends_and_Forecast_2012_-_2018

[B4] Kataoka M Sasaki M Hidalgo AR Nakano M Shimizu S Glycolic acid production using ethylene glycol-oxidizing microorganisms Biosci Biotechnol Biochem 2001 65 10 2265 2270 10.1271/bbb.65.2265 11758919

[B5] He Y-C Xu J-H Su J-H Zhou L Bioproduction of glycolic acid from glycolonitrile with a new bacterial isolate of alcaligenes sp. ECU0401 Appl Biochem Biotechnol 2010 160 5 1428 1440 10.1007/s12010-009-8607-y 19333557

[B6] Ñancucheo I Johnson DB Production of glycolic acid by chemolithotrophic iron- and sulfur-oxidizing bacteria and its role in delineating and sustaining acidophilic sulfide mineral-oxidizing consortia Appl Environ Microbiol 2010 76 2 461 467 10.1128/AEM.01832-09 19933342 PMC2805229

[B7] Kunze M Pracharoenwattana I Smith SM Hartig A A central role for the peroxisomal membrane in glyoxylate cycle function Biochim Biophys Acta 2006 1763 12 1441 1452 10.1016/j.bbamcr.2006.09.009 17055076

[B8] Soucaille P Glycolic Acid Production by Fermentation from Renewable Resources 2007 Patent WO07141316 A2

[B9] Dischert W Soucaille P Method for Producting High Amount of Glycolic Acid by Fermentation 2010 Patent WO10108909 A1

[B10] Dischert W Colomb C Soucaille P Fermentation Process for Producing Glycolic Acid 2011 Patent WO11036213 A2

[B11] Dischert W Figge R Soucaille P Use of Inducible Promoters in the Production of Glycolic Acid 2011 Patent WO11157728 A1

[B12] Martin CH Dhamankar H Tseng H Sheppard MJ Reisch CR Prather KLJ A platform pathway for production of 3-hydroxyacids provides a biosynthetic route to 3-hydroxy-γ-butyrolactone Nat Commun 2013 4 1414 23361005 10.1038/ncomms2418

[B13] Rintala E Pitkänen JP Vehkomäki ML Penttilä M Ruohonen L The ORF *YNL274c* (*GOR1*) codes for glyoxylate reductase in *Saccharomyces cerevisiae* Yeast 2007 24 2 129 136 10.1002/yea.1434 17173333

[B14] Hoover GJ Van Cauwenberghe OR Breitkreuz KE Clark SM Merrill AR Shelp BJ Characteristics of an *Arabidopsis* glyoxylate reductase: general biochemical properties and substrate specificity for the recombinant protein, and developmental expression and implications for glyoxylate and succinic semialdehyde metabolism in planta Can J Bot 2007 85 9 883 895 10.1139/B07-081

[B15] Allan WL Clark SM Hoover GJ Shelp BJ Role of plant glyoxylate reductases during stress: a hypothesis Biochem J 2009 423 1 15 22 10.1042/BJ20090826 19740079 PMC2762691

[B16] Toivari MH Aristidou A Ruohonen L Penttilä M Conversion of xylose to ethanol by recombinant *Saccharomyces cerevisiae*: importance of xylulokinase (*XKS1*) and oxygen availability Metab Eng 2001 3 3 236 249 10.1006/mben.2000.0191 11461146

[B17] Jouhten P Rintala E Huuskonen A Tamminen A Toivari M Wiebe M Ruohonen L Penttilä M Maaheimo H Oxygen dependence of metabolic fluxes and energy generation of *Saccharomyces cerevisiae* CEN.PK113–1A BMC Syst Biol 2008 2 60 10.1186/1752-0509-2-60 18613954 PMC2507709

[B18] Duntze W Neumann D Atzpodien W Holzer H Gancedo JM Studies on the regulation and localization of the glyoxylate cycle enzymes in *Saccharomyces cerevisiae* Eur J Biochem 1969 10 1 83 89 5345986 10.1111/j.1432-1033.1969.tb00658.x

[B19] Kabisch J Pratzka I Meyer H Albrecht D Lalk M Ehrenreich A Schweder T Metabolic engineering of *Bacillus subtilis* for growth on overflow metabolites Micob Cell Fact 2013 12 1 72 10.1186/1475-2859-12-72 PMC3728045 23886069

[B20] Rajgarhia V Koivuranta K Penttilä M Ilmen M Suominen P Aristidou A Miller CK Olson S Ruohonen L Genetically modified yeast species and fermentation processes using genetically modified yeast 2004 Patent WO04099381

[B21] Sauer M Porro D Mattanovich D Branduardi P 16 Years research on lactic acid production with yeast - ready for the market? Biotechnol Genet Eng Rev 2010 27 229 256 10.1080/02648725.2010.10648152 21415900

[B22] Miller C Fosmer A Rush B McMullin T Beacom D Suominen P Editor-in-Chief, Murray M-Y 3.17 - Industrial Production of Lactic Acid Comprehensive Biotechnology 2011 2 Burlington: Academic Press 179 188

[B23] Tochikura T Fukuda H Moriguchi M Purification and properties of glyoxylate reductase I from baker’s yeast J Biochem 1979 86 1 105 110 383706

[B24] Fukuda H Moriguchi M Tochikura T Purification and enzymatic properties of glyoxylate reductase II from baker’s yeast J Biochem 1980 87 3 841 846 6993451 10.1093/oxfordjournals.jbchem.a132814

[B25] Simpson JP Di Leo R Dhanoa PK Allan WL Makhmoudova A Clark SM Hoover GJ Mullen RT Shelp BJ Identification and characterization of a plastid-localized *Arabidopsis* glyoxylate reductase isoform: comparison with a cytosolic isoform and implications for cellular redox homeostasis and aldehyde detoxification J Exp Bot 2008 59 9 2545 2554 10.1093/jxb/ern123 18495639 PMC2423656

[B26] Ching SL Gidda SK Rochon A Van Cauwenberghe OR Shelp BJ Mullen RT Glyoxylate Reductase isoform 1 is localized in the cytosol and Not peroxisomes in plant cells J Integr Plant Biol 2012 54 152 168 10.1111/j.1744-7909.2012.01103.x 22309191

[B27] Nuñez MF Pellicer MT Badia J Aguilar J Baldoma L Biochemical characterization of the 2-ketoacid reductases encoded by *ycdW* and *yiaE* genes in *Escherichia coli* Biochem J 2001 354 Pt 3 707 715 11237876 10.1042/0264-6021:3540707 PMC1221703

[B28] Fauvart M Braeken K Daniels R Vos K Ndayizeye M Noben JP Robben J Vanderleyden J Michiels J Identification of a novel glyoxylate reductase supports phylogeny-based enzymatic substrate specificity prediction Biochim Biophys Acta 2007 1774 9 1092 1098 10.1016/j.bbapap.2007.06.009 17693143

[B29] Ogino H Nakayama H China H Kawata T Doukyu N Yasuda M Characterization of recombinant glyoxylate reductase from thermophile *Thermus thermophilus* HB27 Biotechnol Prog 2008 24 2 321 325 10.1021/bp0702469 18302405

[B30] Ohshima T Nunoura-Kominato N Kudome T Sakuraba H A novel hyperthermophilic archaeal glyoxylate Reductase from *Thermococcus litoralis*. Characterization, gene cloning, nucleotide sequence and expression in *Escherichia coli* Eur J Biochem 2001 268 17 4740 4747 10.1046/j.1432-1327.2001.02394.x 11532010

[B31] Yoshikawa S Arai R Kinoshita Y Uchikubo-Kamo T Wakamatsu T Akasaka R Masui R Terada T Kuramitsu S Shirouzu M Yokoyama S Structure of archaeal glyoxylate reductase from *Pyrococcus horikoshii* OT3 complexed with nicotinamide adenine dinucleotide phosphate Acta Crystallogr D Biol Crystallogr 2007 63 Pt 3 357 365 17327673 10.1107/S0907444906055442

[B32] Mdluli K Booth MP Brady RL Rumsby G A preliminary account of the properties of recombinant human Glyoxylate reductase (GRHPR), LDHA and LDHB with glyoxylate, and their potential roles in its metabolism Biochim Biophys Acta 2005 1753 2 209 216 10.1016/j.bbapap.2005.08.004 16198644

[B33] Herrero P Fernández R Moreno F Differential sensitivities to glucose and galactose repression of gluconeogenic and respiratory enzymes from *Saccharomyces cerevisiae* Arch Microbiol 1985 143 3 216 219 10.1007/BF00411238 3006623

[B34] López-Boado YS Herrero P Fernández T Fernández R Moreno F Glucose-stimulated phosphorylation of yeast isocitrate lyase in vivo J Gen Microbiol 1988 134 9 2499 2505 3076186 10.1099/00221287-134-9-2499

[B35] Ordiz I Herrero P Rodicio R Moreno F Glucose-induced inactivation of isocitrate lyase in *Saccharomyces cerevisiae* is mediated by the cAMP-dependent protein kinase catalytic subunits Tpk1 and Tpk2 FEBS Lett 1996 385 1–2 43 46 8641464 10.1016/0014-5793(96)00344-4

[B36] López-Boado YS Herrero P Gascón S Moreno F Catabolite inactivation of isocitrate lyase from *Saccharomyces cerevisiae* Arch Microbiol 1987 147 3 231 234 10.1007/BF00463480 3036035

[B37] Gangloff SP Marguet D Lauquin GJ Molecular cloning of the yeast mitochondrial aconitase gene (*ACO1*) and evidence of a synergistic regulation of expression by glucose plus glutamate Mol Cell Biol 1990 10 7 3551 3561 1972545 10.1128/mcb.10.7.3551-3561.1990 PMC360790

[B38] Kim KS Rosenkrantz MS Guarente L *Saccharomyces cerevisiae* contains two functional citrate synthase genes Mol Cell Biol 1986 6 6 1936 1942 3023912 10.1128/mcb.6.6.1936-1942.1986 PMC367731

[B39] Hartig A Simon MM Schuster T Daugherty JR Yoo HS Cooper TG Differentially regulated malate synthase genes participate in carbon and nitrogen metabolism of *S. cerevisiae* Nucleic Acids Res 1992 20 21 5677 5686 10.1093/nar/20.21.5677 1454530 PMC334402

[B40] Minard KI McAlister-Henn L Isolation, nucleotide sequence analysis, and disruption of the *MDH2* gene from *Saccharomyces cerevisiae*: evidence for three isozymes of yeast malate dehydrogenase Mol Cell Biol 1991 11 1 370 380 1986231 10.1128/mcb.11.1.370-380.1991 PMC359635

[B41] López ML Redruello B Valdés E Moreno F Heinisch JJ Rodicio R Isocitrate lyase of the yeast *Kluyveromyces lactis* is subject to glucose repression but not to catabolite inactivation Curr Genet 2004 44 6 305 316 10.1007/s00294-003-0453-9 14569415

[B42] Hardie DG Carling D Carlson M The AMP-activated/SNF1 protein kinase subfamily: metabolic sensors of the eukaryotic cell? Annu Rev Biochem 1998 67 821 855 10.1146/annurev.biochem.67.1.821 9759505

[B43] Charbon G Breunig KD Wattiez R Vandenhaute J Noël-Georis I Key role of Ser562/661 in Snf1-dependent regulation of Cat8p in *Saccharomyces cerevisiae* and *Kluyveromyces lactis* Mol Cell Biol 2004 24 10 4083 4091 10.1128/MCB.24.10.4083-4091.2004 15121831 PMC400452

[B44] Jiang R Carlson M Glucose regulates protein interactions within the yeast SNF1 protein kinase complex Genes Dev 1996 10 24 3105 3115 10.1101/gad.10.24.3105 8985180

[B45] Sanz P Alms GR Haystead TA Carlson M Regulatory interactions between the Reg1-Glc7 protein phosphatase and the Snf1 protein kinase Mol Cell Biol 2000 20 4 1321 1328 10.1128/MCB.20.4.1321-1328.2000 10648618 PMC85274

[B46] Ludin K Jiang R Carlson M Glucose-regulated interaction of a regulatory subunit of protein phosphatase 1 with the Snf1 protein kinase in *Saccharomyces cerevisiae* Proc Natl Acad Sci U S A 1998 95 11 6245 6250 10.1073/pnas.95.11.6245 9600950 PMC27646

[B47] Turcotte B Liang XB Robert F Soontorngun N Transcriptional regulation of nonfermentable carbon utilization in budding yeast FEMS Yeast Res 2010 10 1 2 13 10.1111/j.1567-1364.2009.00555.x 19686338 PMC5003605

[B48] Kunze M Kragler F Binder M Hartig A Gurvitz A Targeting of malate synthase 1 to the peroxisomes of *Saccharomyces cerevisiae* cells depends on growth on oleic acid medium Eur J Biochem 2002 269 3 915 922 10.1046/j.0014-2956.2001.02727.x 11846793

[B49] Vélot C Lebreton S Morgunov I Usher KC Srere PA Metabolic effects of mislocalized mitochondrial and peroxisomal citrate synthases in yeast *Saccharomyces cerevisiae* Biochemistry 1999 38 49 16195 16204 10.1021/bi991695n 10587442

[B50] De Jong-Gubbels P Bauer J Niederberger P Stückrath I Kötter P Van Dijken JP Pronk JT Physiological characterisation of a pyruvate-carboxylase-negative *Saccharomyces cerevisiae* mutant in batch and chemostat cultures Antonie Van Leeuwenhoek 1998 74 4 253 263 10.1023/A:1001772613615 10081585

[B51] Heinisch JJ Buchwald U Gottschlich A Heppeler N Rodicio R A tool kit for molecular genetics of *Kluyveromyces lactis* comprising a congenic strain series and a set of versatile vectors FEMS Yeast Res 2010 10 3 333 342 10.1111/j.1567-1364.2009.00604.x 20522115

[B52] Verho R Putkonen M Londesborough J Penttilä M Richard P A novel NADH-linked L-xylulose reductase in the L-arabinose catabolic pathway of yeast J Biol Chem 2004 279 15 14746 14751 10.1074/jbc.M312533200 14736891

[B53] Gietz RD Schiestl RH High-efficiency yeast transformation using the LiAc/SS carrier DNA/PEG method Nat Protocols 2007 2 1 31 34 10.1038/nprot.2007.13 17401334

[B54] Güldener U Heck S Fielder T Beinhauer J Hegemann JH A new efficient gene disruption cassette for repeated use in budding yeast Nucleic Acids Res 1996 24 13 2519 2524 10.1093/nar/24.13.2519 8692690 PMC145975

[B55] Gueldener U Heinisch J Koehler GJ Voss D Hegemann JH A second set of *loxP* marker cassettes for Cre-mediated multiple gene knockouts in budding yeast Nucleic Acids Res 2002 30 6 e23 10.1093/nar/30.6.e23 11884642 PMC101367

[B56] Colot HV Park G Turner GE Ringelberg C Crew CM Litvinkova L Weiss RL Borkovich KA Dunlap JC A high-throughput gene knockout procedure for *Neurospora* reveals functions for multiple transcription factors Proc Natl Acad Sci U S A 2006 103 27 10352 10357 10.1073/pnas.0601456103 16801547 PMC1482798

[B57] Winston F Dollard C Ricupero-Hovasse SL Construction of a set of convenient *Saccharomyces cerevisiae* strains that are isogenic to S288C Yeast 1995 11 1 53 55 10.1002/yea.320110107 7762301

[B58] Sherman F Fink G Hicks JB Methods in Yeast Genetics. A Laboratory Manual Cold Springs Harbor 1983 New York: Cold Springs Harbor Laboratory

[B59] Christianson TW Sikorski RS Dante M Shero JH Hieter P Multifunctional yeast high-copy-number shuttle vectors Gene 1992 110 1 119 122 10.1016/0378-1119(92)90454-W 1544568

[B60] Boles E Göhlmann HWH Zimmermann FK Cloning of a second gene encoding 6-phosphofructo-2-kinase in yeast, and characterization of mutant strains without fructose-2,6-bisphosphate Mol Microbiol 1996 20 1 65 76 10.1111/j.1365-2958.1996.tb02489.x 8861205

[B61] Isocitrate Lyase Assay in Yeast http://biopuce.insa-toulouse.fr/jmflab/protocole/Isocitratelyaseassayinyeast.htm

[B62] Tohmola N Ahtinen J Pitkänen J Parviainen V Joenväärä S Hautamäki M Lindroos P Mäkinen J Renkonen R On-line high performance liquid chromatography measurements of extracellular metabolites in an aerobic batch yeast (*Saccharomyces cerevisiae*) culture Biotechnol Bioprocess Eng 2011 16 2 264 272 10.1007/s12257-010-0147-3

[B63] Turkia H Sirén H Pitkänen J Wiebe M Penttilä M Capillary electrophoresis for the monitoring of carboxylic acid production by *Gluconobacter oxydans* J Chromatogr A 2010 1217 9 1537 1542 10.1016/j.chroma.2009.12.075 20074741

